# Mitochondrial DNA damage in HIV infection: a mechanistic driver of immunometabolic dysfunction and chronic inflammation

**DOI:** 10.3389/fimmu.2025.1722463

**Published:** 2026-01-12

**Authors:** Li Ma, Xiaolei Wang, Huanbin Xu

**Affiliations:** 1 The Division of Comparative Pathology, Tulane National Biomedical Research Center (TNBRC), Covington, LA, United States; 2 The Division of Comparative Pathology, Department of Pathology and Laboratory Medicine, Tulane Celia Scott Weatherhead School of Medicine, New Orleans, LA, United States; 3 Tulane University School of Public Health and Tropical Medicine, New Orleans, LA, United States

**Keywords:** antiretroviral therapy, cGAS-STING, HIV, immunometabolism, inflammaging, mtDNA, oxidative stress

## Abstract

Mitochondria are central regulators of cellular metabolism and immunity. Human immunodeficiency virus (HIV) infection and antiretroviral therapy (ART) are associated with metabolic complications and chronic inflammation, yet the underlying mechanisms remain incompletely understood. Increasing evidence implicates mitochondrial dysfunction—particularly mitochondrial DNA (mtDNA) damage—as a key contributor. HIV/SIV infection and ART both compromise mtDNA integrity through direct and indirect mechanisms, leading to impaired oxidative phosphorylation, dysregulated reactive oxygen species, and altered mitochondrial dynamics. These changes contribute to immune cell bioenergetic failure, T cell exhaustion, and cytosolic release of mtDNA, which can activate cGAS-STING and NLRP3 pathways to sustain chronic inflammation. In addition, certain ART drugs, especially early nucleoside reverse transcriptase inhibitors, inhibit polymerase γ, driving mtDNA depletion and mutation accumulation that underlie toxicities such as lipodystrophy, neuropathy, and accelerated aging. Monitoring mtDNA copy number and mutational burden may offer useful biomarkers of immune recovery and treatment-related complications. Targeting mitochondrial protection and repair represents a promising strategy to improve long-term outcomes in people living with HIV.

## 1 Introduction

The human immunodeficiency virus (HIV) pandemic, now in its fifth decade, has been transformed by the remarkable success of antiretroviral therapy (ART). ART suppresses viral replication to undetectable levels, restoring near-normal life expectancy for people living with HIV (PLWH) (1, 2). However, this success has unveiled a new set of challenges. Despite effective virological control, PLWH experience a higher incidence of age-related comorbidities, including cardiovascular disease, metabolic syndrome, neurocognitive disorders, and non-AIDS-defining cancers (3–5). This phenomenon is driven by a persistent state of chronic immune activation and inflammation—often referred to as “inflammaging” —the origins of which are multifactorial and not fully elucidated (6, 7).

At the cellular heart of this problem lies immunometabolism, the intricate interplay between metabolic pathways and immune cell function. Immune cells dynamically reprogram their metabolic states to support their specific functions, whether it’s the rapid proliferation of effector cells or the long-term persistence of memory cells (8, 9). Mitochondria, the quintessential metabolic organelles, are master regulators of this process. Beyond their canonical role in producing adenosine triphosphate (ATP) through oxidative phosphorylation (OXPHOS), mitochondria are integral to biosynthetic pathways, redox balance, calcium signaling, apoptosis, and serve as signaling platforms for innate immune responses (10–13).

Mitochondria contain their own multi-copy circular genome (mtDNA), which encodes 13 essential subunits of the OXPHOS system as well as 22 tRNAs and 2 rRNAs required for their translation (14). The integrity of mtDNA is therefore paramount for cellular energy homeostasis. However, mtDNA is particularly vulnerable to damage due to its proximity to the ROS-generating electron transport chain (ETC), lack of protective histones, and relatively inefficient repair mechanisms (15, 16).

Both HIV infection itself and certain ART drugs can inflict damage on mitochondria. HIV proteins such as Vpr and Tat have been shown to directly impair mitochondrial function, induce ROS production, and promote mtDNA damage (17–20). Concurrently, some NRTIs inhibit mitochondrial DNA polymerase γ, leading to mtDNA depletion and the accumulation of deletions and point mutations (21–23). This mitochondrial dysfunction manifests as a bioenergetic crisis that forces immune cells towards inefficient glycolytic metabolism, impairing effector functions, and promoting apoptosis. Furthermore, damaged mitochondria can release mtDNA into the cytosol, where it is recognized by pattern-recognition receptors such as cGAS, triggering STING-dependent type I interferon and pro-inflammatory cytokine production, thereby fueling the chronic inflammation observed in PLWH (10, 24, 25).

It is crucial to recognize that chronic inflammation and immune dysfunction in PLWH are multifactorial and extend beyond mitochondrial dysregulation. Well-established contributors include microbial translocation due to gut barrier impairment, residual viral replication in sanctuary sites, reactivation of latent co-infections (e.g., cytomegalovirus), and increased cellular senescence (26–28). These processes do not operate in isolation; instead, they engage in complex crosstalk with mitochondrial pathways. For instance, systemic inflammation driven by microbial products can exacerbate mitochondrial oxidative stress, whereas mitochondrial dysfunction—via impaired ATP production and release of damage-associated molecular patterns (DAMPs)—can itself amplify inflammatory responses and worsen tissue injury (29, 30). Thus, mitochondrial integrity sits at a critical intersection, acting both as a target of upstream insults and as a downstream amplifier of the immunopathogenic cascade. This review focuses specifically on the role of mtDNA damage within this network, synthesizing evidence for its function as a mechanistic driver and an actionable therapeutic target in the immunometabolic complications of HIV infection.

This review aims to synthesize current knowledge on how HIV/SIV infection and ART induce mtDNA damage and how this damage acts as a key indicator and mechanistic driver of associated immunometabolic changes. We will examine the consequences of mtDNA dysfunction for immune cell fate and systemic inflammation, discuss the clinical relevance of mtDNA as a biomarker for toxicity and disease progression, and highlight emerging therapeutic strategies aimed at preserving mitochondrial health to improve the long-term well-being of PLWH.

## 2 Mitochondrial fundamentals: from energy hub to immune regulator

Mitochondria are often termed the “powerhouses” of the cell due to their quintessential role in energy production. However, their function extends far beyond adenosine triphosphate (ATP) generation, encompassing critical roles in biosynthetic processes, cell signaling, and the regulation of programmed cell death. In the context of immunity, mitochondria have emerged as central hubs that integrate metabolic and immunological signals, thereby orchestrating appropriate immune cell responses.

### 2.1 Structure, genome, and basic functions of mitochondria

The mammalian mitochondrial genome is a multi-copy, circular DNA molecule of approximately 16.6 kilobaseshoused within the mitochondrial matrix. Unlike nuclear DNA, mtDNA lacks protective histones and has limited DNA repair capacity, rendering it particularly vulnerable to genotoxic stress, such as from reactive oxygen species (ROS) generated during oxidative phosphorylation (OXPHOS) (31).

The mtDNA encodes 37 genes essential for mitochondrial function: 13 core subunits of the OXPHOS complexes, along with 22 transfer RNAs (tRNAs) and 2 ribosomal RNAs (rRNAs) required for their intramitochondrial translation (14). It is critical to note that the vast majority of mitochondrial proteins (over 1,000) are encoded by the nuclear genome. These include all factors necessary for mtDNA replication, transcription, translation, and repair (e.g., DNA polymerase γ, TFAM), as well as those required for the proper assembly of the OXPHOS system (14, 32, 33). This dual genetic control necessitates exquisite coordination between the nucleus and mitochondria to maintain cellular homeostasis.

The primary function of mitochondria is the production of ATP through OXPHOS. This process couples electron transfer through the electron transport chain (ETC) to the pumping of protons across the inner mitochondrial membrane, creating an electrochemical gradient. The energy stored in this gradient is then harnessed by ATP synthase (Complex V) to phosphorylate ADP into ATP (34, 35). The reducing equivalents (NADH and FADH2) that drive this process are largely generated by the tricarboxylic acid (TCA) cycle from nutrients such as glucose and fatty acids.

**Figure 1:**
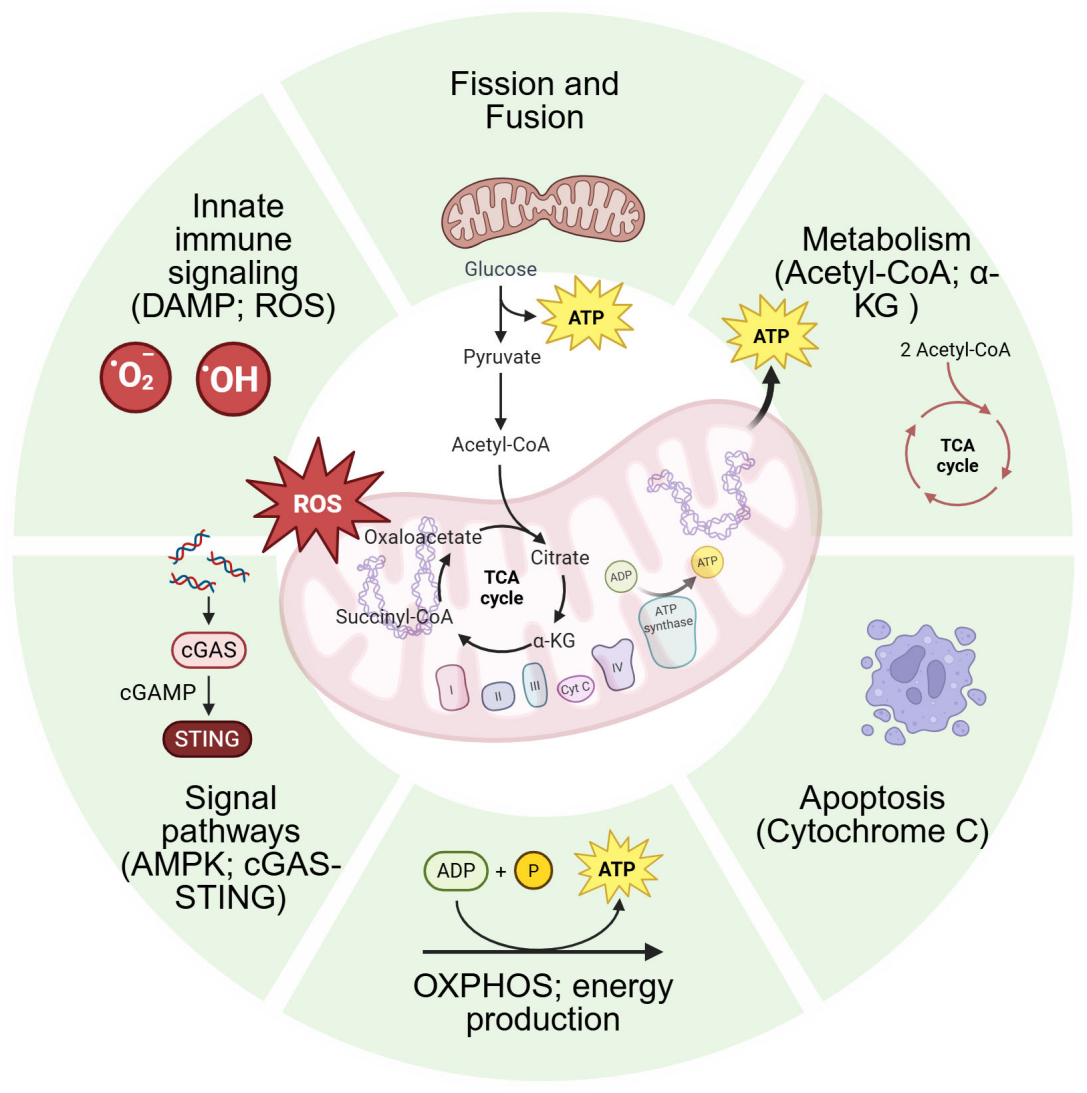
Schematic diagram of mitochondrial structure and multifunctionality. This diagram depicts the key anatomical structures of mitochondria, including the outer and inner membranes, cristae, and matrix. It also shows the circular mitochondrial DNA (mtDNA), the electron transport chain, and the tricarboxylic acid (TCA) cycle. The surrounding icons represent its core functions: energy production (ATP generation via oxidative phosphorylation), central metabolism (the TCA cycle), signal transduction (e.g., AMPK and cGAS-STING pathways), dynamics (fission and fusion), innate immune activation (via DAMPs and ROS), and regulation of cell fate (e.g., cytochrome c release during apoptosis). This figure provides a foundational visual summary of the mitochondrial roles discussed in the text. Figures were created with BioRender.com.

Beyond energy conversion, mitochondria are indispensable for a myriad of other cellular processes, including heme and steroid hormone synthesis, calcium ion (Ca^2+^) buffering to regulate intracellular signaling, and the initiation of apoptosis via the mitochondrial pathway (36). A summary of the mitochondrial structure and its key functional landscapes is provided in **Figure 1**.

### 2.2 The central role of mitochondria in cellular immunometabolism

Immunometabolism investigates the intricate link between metabolic pathways and immune cell function. The activation, differentiation, and effector functions of immune cells are energetically and biosynthetically demanding processes that rely on dynamic metabolic reprogramming, heavily dependent on mitochondrial function. Mitochondria serve as central regulators in this process through several key mechanisms:

#### 2.2.1 Metabolic substrate provision

Mitochondria provide not only ATP but also intermediate metabolites from the TCA cycle (e.g., citrate, succinate, α-ketoglutarate) that serve as precursors for the biosynthesis of lipids, proteins, and nucleic acids, which are crucial for rapid immune cell proliferation and cytokine production (8, 9).

#### 2.2.2 Signal integration via metabolites

Mitochondrial metabolites act as signaling molecules that influence immune cell fate and function through epigenetic and post-translational modifications. **Succinate**: Accumulation stabilizes Hypoxia-Inducible Factor-1α (HIF-1α), driving pro-inflammatory gene expression in macrophages (e.g., IL-1β) (37). **α-Ketoglutarate (α-KG)**: Acts as a cofactor for dioxygenases like TET enzymes and JMJD histone demethylases, influencing the epigenetic landscape and promoting anti-inflammatory phenotypes, such as regulatory T cell (Treg) differentiation (38). **Acetyl-CoA**: Serves as the substrate for protein acetylation, including histone acetylation, which opens chromatin and facilitates the transcriptional programs necessary for immune cell activation (39).

#### 2.2.3 Reactive oxygen species signaling

While excessive ROS causes oxidative damage, physiological levels of mitochondrial ROS (mtROS) are crucial second messengers that activate key signaling pathways like NF-κB and NLRP3 inflammasome, amplifying inflammatory responses and enhancing antimicrobial activity in phagocytes (40, 41).

#### 2.2.4 Regulation of apoptosis and innate immune signaling

Mitochondria control the intrinsic apoptotic pathway by releasing Cytochrome C. Furthermore, the mitochondrial outer membrane provides a platform for the assembly of signaling complexes like the mitochondrial antiviral-signaling (MAVS) protein, which is critical for initiating robust antiviral interferon responses upon viral RNA detection (42–44).

Mitochondria are not passive energy factories but dynamic, signaling organelles that interpret metabolic cues to dictate immune cell responses. Their central role in immunometabolism makes them a critical target for dysregulation by pathogens like HIV, leading to the profound immune and metabolic disturbances observed in infection.

## 3 Mechanisms of HIV/SIV-induced mitochondrial dysfunction

HIV/SIV infection instigates a profound disruption of mitochondrial physiology that extends beyond mere bioenergetic failure to encompass widespread immunometabolic dysregulation. This dysfunction is not a passive consequence but an active process whereby viral components and the resulting inflammatory milieu directly impair mitochondrial integrity, fueling a vicious cycle of immune exhaustion and viral persistence.

### 3.1 Metabolic reprogramming of immune cells in HIV/SIV infection

**Figure 2:**
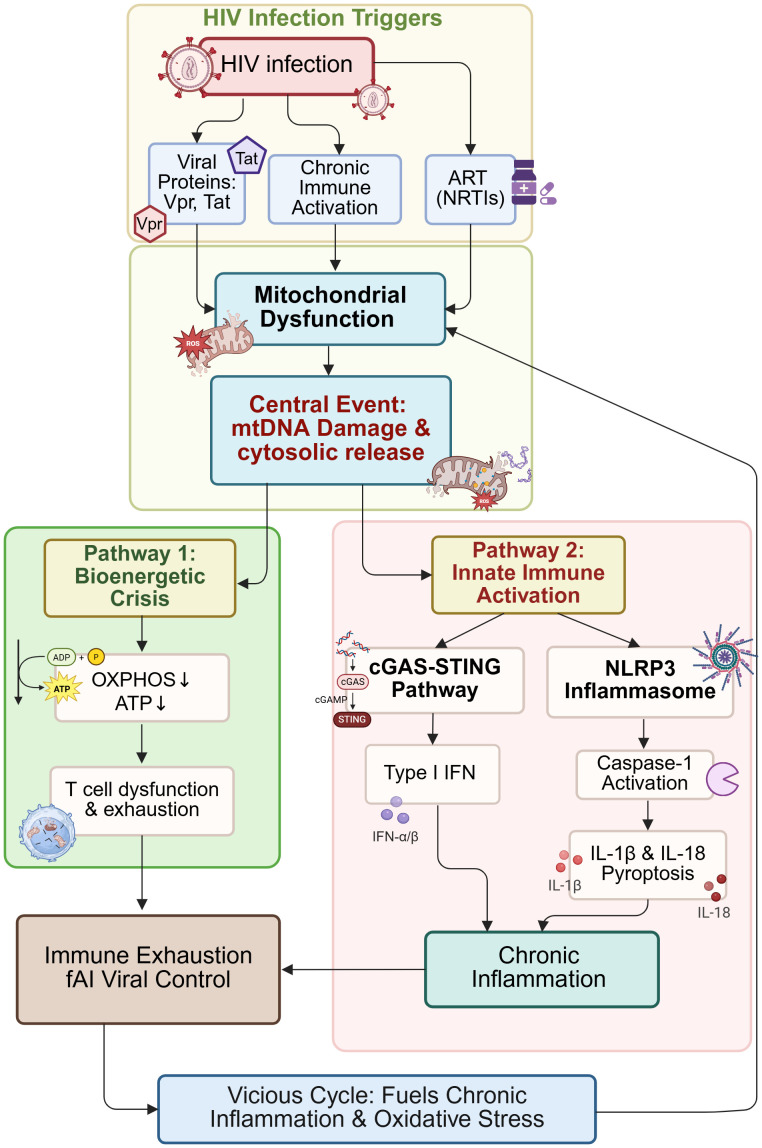
Metabolic reprogramming of key immune cells during HIV/SIV infection. This schematic contrasts the metabolic preferences of major immune cell subsets under homeostatic conditions (Left) and their dysregulation during HIV/SIV infection (Right). In health, naïve T cells rely on oxidative phosphorylation (OXPHOS) and fatty acid oxidation (FAO); upon activation, effector T cells shift to glycolysis, while memory T cells revert to OXPHOS for long-term persistence. M1 macrophages utilize glycolysis, and M2 macrophages depend on OXPHOS/FAO. HIV/SIV infection induces profound metabolic alterations: CD4+ T cells exhibit diminished OXPHOS and an aberrant, hyperactive glycolytic state reminiscent of the Warburg effect. CD8+ T cells display a global metabolic exhaustion, with reductions in both OXPHOS and glycolysis. Macrophages undergo a metabolic shift towards enhanced glycolysis and impaired OXPHOS/FAO, accompanied by intracellular droplet accumulation, driving them towards a foamy phenotype. Figures were created with BioRender.com.

A hallmark of HIV pathogenesis is the profound metabolic reprogramming of key immune cells. As illustrated in **Figure 2**, this reprogramming shifts cells from their normal metabolic states toward maladaptive profiles that support viral persistence rather than effective immunity.

#### 3.1.1 Metabolism of immune cells in homeostasis

Under homeostatic conditions, immune cells adopt distinct metabolic configurations tailored to their functions. Naive T cells primarily rely on oxidative phosphorylation (OXPHOS) coupled with fatty acid oxidation (FAO) to meet their energy demands for long-term survival and vigilance. This catabolic metabolism generates ample ATP efficiently and supports a quiescent state. Upon antigen encounter, successful activation triggers a metabolic shift: Effector T cells upregulate aerobic glycolysis (the Warburg effect) to rapidly generate biosynthetic precursors (nucleotides, amino acids, lipids) necessary for clonal expansion and cytokine production, while still maintaining OXPHOS capacity. As the infection is cleared, a subset of these cells differentiates into Memory T cells, which revert to a reliance on OXPHOS and FAO, allowing for long-term persistence and rapid recall (45–51). Similarly, in macrophages, classical activation of pro-inflammatory M1 macrophages promotes glycolysis to support antimicrobial function and cytokine production, while anti-inflammatory M2 macrophages primarily utilize OXPHOS and FAO for tissue repair and homeostasis (52, 53).

#### 3.1.2 HIV/SIV-induced pathogenic reprogramming

HIV/SIV infection catastrophically disrupts these finely tuned metabolic programs, leading to exhaustion and dysfunction. CD4+ T Cells: HIV-1 infection dramatically reprograms CD4+ T cell metabolism, upregulating nearly all branches of metabolism, including glucose, lipid, and amino acid metabolism, to fuel its own replication (54). This virus-driven hypermetabolic state is paradoxically coupled with impaired mitochondrial OXPHOS capacity. The resulting energy deficit, combined with the virus’s massive demand for biosynthetic precursors, leads to a state of metabolic exhaustion. Cells become abnormally and inefficiently dependent on aerobic glycolysis (the Warburg effect), which cannot sustain long-term survival, ultimately promoting activation-induced apoptosis and the collapse of the CD4+ T cell pool (54, 55). CD8+ T Cells: The fate of virus-specific CD8+ T cells during chronic HIV/SIV infection is a key driver of disease progression. In stark contrast to the potent glycolytic-effector phenotype seen in acute infection, upon persistent antigenic stimulation, these cells undergo a distinct process of exhaustion. A very early feature of this exhaustion is bioenergetic insufficiency, characterized by restricted glucose uptake and utilization despite ongoing mTOR signaling (56). This results in severely impaired glycolytic and oxidative metabolic fluxes. The suppression of mitochondrial OXPHOS, particularly ADP-coupled respiration, is sufficient to block proliferation by limiting nucleotide triphosphate synthesis and upregulate exhaustion markers (57). This metabolic paralysis is a fundamental barrier to effective cytotoxic function (56, 58). Macrophages: HIV infection skews macrophages towards a sustained pro-inflammatory M1-like phenotype. These cells exhibit heightened glycolytic flux, which supports the production and release of pro-inflammatory cytokines like IL-1β and TNF-α. This shift, while part of an antiviral response, contributes to chronic tissue inflammation and damage. Furthermore, HIV impairs mitochondrial fatty acid oxidation (FAO) in these cells, leading to intracellular lipid accumulation and potentially promoting atherosclerotic plaque formation (59–61).

This widespread pathogenic reprogramming towards inefficient glycolysis, in the face of mitochondrial dysfunction, creates a pro-inflammatory environment and deprives immune cells of the sustained energy required for memory formation and effective viral control.

### 3.2 Mitochondrial metabolites as immune signaling molecules

In HIV infection, mitochondrial dysfunction alters the levels of key metabolic intermediates, converting them from homeostatic signaling molecules into drivers of pathology. TCA Cycle Disruption and Epigenetic Alterations: HIV-induced mitochondrial stress reduces metabolites such as acetyl-CoA and α-ketoglutarate (α-KG). Lower acetyl-CoA availability can limit histone acetylation, repressing the transcription of genes essential for immune function. More critically, a decrease in α-KG-a necessary cofactor for ten-eleven translocation (TET) DNA demethylases and Jumonji-domain (JMJD) histone demethylases-leads to hypermethylation of DNA and histones. This epigenetic silencing can lock T cells into an exhausted state and impair memory T cell differentiation, weakening long-term immunity (62, 63).

#### 3.2.1Succinate Accumulation and HIF-1α Stabilization

Damage to the ETC, particularly at Complex II (succinate dehydrogenase), can lead to the accumulation of succinate. In macrophages, elevated succinate inhibits prolyl hydroxylases (PHDs), leading to the stabilization of the transcription factor HIF-1α. This promotes a potent pro-inflammatory gene expression profile, enhancing glycolysis and the production of IL-1β, thereby exacerbating chronic inflammation (37, 64).

#### 3.2.2 Lactate and acidic microenvironment

Excessive glycolysis, a consequence of failed OXPHOS, results in massive lactate production. This acidifies the tissue microenvironment, which can suppress T cell function and cytotoxicity, further impairing the antiviral immune response (65).

### 3.3 Disruption of mitochondrial dynamics and quality control

Mitochondria are highly dynamic organelles that undergo constant fission (division) and fusion (merging) to maintain a healthy network. HIV infection severely disrupts this balance and overwhelms quality control mechanisms. Imbalanced Dynamics: Excessive Fission- HIV proteins, such as Vpr, promote a shift towards mitochondrial fission. This is mediated through the upregulation and activation of the fission protein DRP1 (17). The resulting fragmented, punctate mitochondria are inefficient for energy production, produce more ROS, and are more prone to permeability transition pore (mPTP) opening. While fission is a normal mechanism for isolating damaged portions for removal, its chronic activation leads to a preponderance of dysfunctional organelles. Suppressed Fusion: Concurrently, HIV infection often downregulates the key proteins mediating mitochondrial fusion, such as MFN1, MFN2 (Mitofusin 1 and Mitofusin 2, regulating outer membrane fusion), and OPA1,(Optic atrophy 1, regulating inner membrane fusion) (17). This loss of fusion prevents the complement of damaged mitochondria with healthy components, further compromising the entire network’s bioenergetic capacity and stability. Failure of Mitophagy: The clearance of damaged, fissioned mitochondria is accomplished through a selective form of autophagy called mitophagy, primarily governed by the PINK1(PTEN-induced kinase 1)-Parkin pathway (66, 67). HIV infection interferes with this critical quality control process. Impaired mitophagy leads to the accumulation of dysfunctional mitochondria that leak ROS and pro-apoptotic factors like Cytochrome C. This not only accelerates cell death but also contributes to the activation of the NLRP3 inflammasome, leading to excessive IL-1β and IL-18 production and driving further inflammation (68, 69).

HIV infection orchestrates a multi-faceted attack on mitochondrial biology. It enforces maladaptive metabolic reprogramming, corrupts mitochondrial signaling metabolites to promote exhaustion and inflammation, and disrupts dynamic balance and quality control systems, leading to the accumulation of damaged organelles. This collective mitochondrial failure is a cornerstone of the immunopathogenesis of HIV/AIDS.

## 4 Mitochondrial DNA: a key player and victim in HIV pathogenesis

Mitochondrial DNA (mtDNA) occupies a dual role in HIV pathogenesis: it is both a target for viral-induced damage and a potent activator of innate immune pathways that drive chronic inflammation. The vulnerability of mtDNA to insult, coupled with its bacterial-like molecular patterns, makes it a central actor in the cycle of metabolic failure and immune dysfunction that characterizes HIV infection.

### 4.1 mtDNA-triggered innate immune signaling pathways (cGAS-STING, NLRP3)

The release of mtDNA into the cytoplasm or extracellular space is a critical event that amplifies inflammation during HIV infection. Mitochondrial damage—caused by membrane depolarization, mPTP (Mitochondrial permeability transition pore) opening, or BAK/BAX (BCL-2 homologous antagonist/killer and BCL-2-associated X protein) macropores formation — allows mtDNA to escaple into the cytosol, where it functions as a potent DAMP (Damage-associated molecular pattern) (70, 71). Cytosolic mtDNA activates the cGAS-STING pathway: cGAS (Cyclic GMP-AMP synthase), generates the second messenger 2’3’-cGAMP, which binds STING (Stimulator of interferon genes) (68). STING then recruits TBK1 (TANK-binding kinase 1), which phosphorylates IRF3 (Interferon regulatory factor 3)), leading to the transcription and production of IFN-α/β (Type I interferons) and other ISGs (Interferon-stimulated genes) (25, 70–76). In chronic HIV infection, persistent mtDNA leakage sustains this pathway, contributing to the elevated IFN-inflammatory signature characteristic of individuals even on ART. Activation, mtDNA, particularly in its oxidized form (ox-mtDNA), can also directly activate the NLRP3 inflammasome, promoting caspase-1 activation, maturation of IL-1β and IL-18, and GSDMD (Gasdermin D)-mediated pyroptosis, thereby fueling chronic inflammation (29, 77, 78). Additionally, mtDNA can engage other innate immune sensors such as AIM2 (Absent in melanoma 2) and TLR9 (Toll-like receptor 9) (when internalized into endosomes), further amplifying the inflammatory responses (79–81). Thus, mtDNA released from damaged mitochondria serves as a central driver of innate immune activation and chronic inflammation in HIV infection.

### 4.2 Mechanisms of HIV/SIV-induced mtDNA damage and dysfunction

HIV uses both direct and indirect mechanisms that compromise mtDNA integrity and mitochondrial function. Vpr (Viral protein R) localizes to mitochondria, where it induces membrane depolarization, increases ROS generation, and promotes mitochondrial mPTP opening. This oxidative environment causes mtDNA lesions, mutations, and deletions (17, 82). **Tat** (transactivator of transcription) protein, even in the absence of productive infection, can enter mitochondria and inhibit the expression and function of TFAM (Mitochondrial transcription factor A), thereby impairing mtDNA replication and transcription and reducing the expression of mtDNA-encoded ETC subunits (18, 19). Indirect Mechanisms further amplify mtDNA damage. Oxidative Stress, driving by ETC dysfunction, NADPH oxidase activation, and chronic immune activation, exposes mtDNA to excessive ROS, resulting in oxidative modifications such as 8-oxo-dG, strand breaks, and mutations (83, 84). Inflammation cytokines (e.g., TNF-α, IL-1β) abundant during HIV infection exacerbate mitochondrial dysfunction and ROS production, forming a self-amplifying loop that increases mtDNA damage (85).

### 4.3 Consequences of mtDNA damage: from bioenergetic crisis to immune exhaustion

mtDNA mutations and depletion impair the synthesis of essential ETC components, resulting in defective OXPHOS, reduced ATP synthesis, collapse of the mitochondrial membrane potential (ΔΨm), and accumulation of metabolic intermediates due to TCA cycle disruption and compensatory glycolysis. Immune cells-particularly CD8+ T cells-are highly dependent on mitochondrial ATP supply, and this bioenergetic failure limits their proliferation, cytokine production, and cytotoxic activity, contributing to T cell exhaustion (86, 87). Loss of ΔΨm also favors mPTP opening () and the release of Cytochrome C, inducing caspase-mediated apoptosis and accelerating immune cells loss (88, 89).

As noted earlier, damaged or released mtDNA activates innate immune pathways such as cGAS-STING and NLRP3, sustaining chronic inflammation and inflammaging (6, 81, 90). This creates a self-reinforcing cycle in which inflammation damages mitochondria, leading to further mtDNA release. Tissues that rely heavily on oxidative metabolism are particularly vulnerable to mtDNA damage. For instance, neurons have high energy demands, and mtDNA damage in the CNS contributes to HIV-associated neurocognitive disorders (HAND) through neuronal energy failure and neuroinflammation (91–95). Similarly, in endothelial cells and cardiomyocytes, mtDNA dysfunction exacerbates oxidative stress and vascular inflammation, accelerating atherosclerosis and increasing cardiovascular risk (96). mtDNA depletion in peripheral nerves and adipose tissue is also linked to classic antiretroviral therapy toxicities such as peripheral neuropathy and lipodystrophy (97–99).

**Figure 3:**
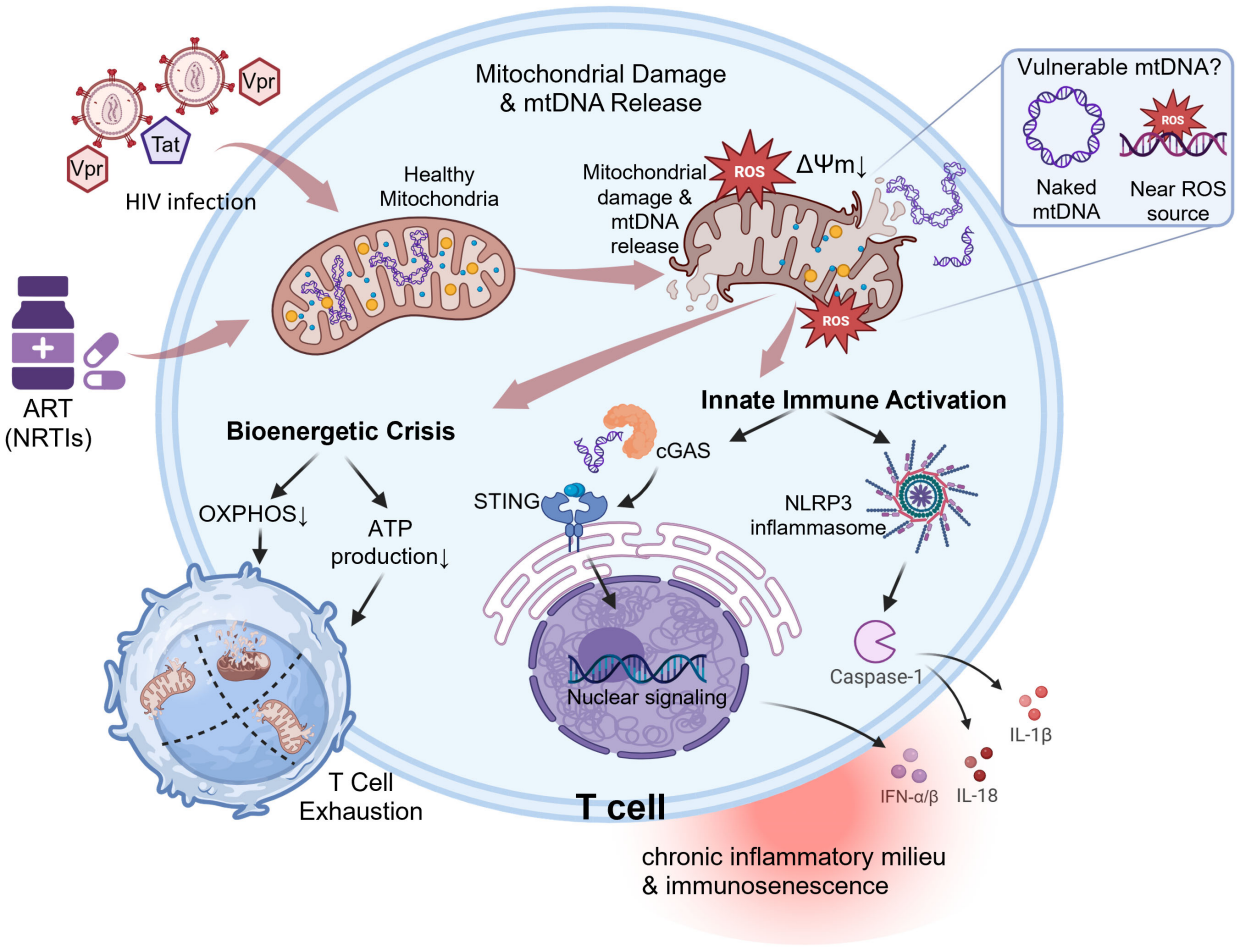
Proposed model linking HIV infection, mitochondrial DNA damage, and T cell exhaustion. HIV infection and its associated factors—including viral proteins (Vpr, Tat), chronic immune activation, and antiretroviral therapy (ART) toxicity—act as instigators of mitochondrial dysfunction and oxidative stress. These insults are particularly detrimental to mitochondrial DNA (mtDNA), which is inherently vulnerable due to its lack of histone protection and high mutation rate. The central event in this model is the damage and subsequent release of mtDNA into the cytosol. Cytosolic mtDNA then triggers two major consequence pathways: (Left Pathway) Bioenergetic Crisis: The loss of mtDNA integrity impairs OXPHOS, leading to a critical depletion of ATP. This energy crisis directly compromises T cell metabolic fitness, proliferation, and effector functions. (Right Pathway) Innate Immune Activation: The released mtDNA is recognized by innate immune sensors. It activates the cGAS-STING pathway, leading to the production of Type I interferons (IFNs), and the NLRP3 inflammasome, resulting in the maturation and release of pro-inflammatory cytokines IL-1β and IL-18. This creates a sustained inflammatory state. The convergence of bioenergetic failure and chronic inflammation drives the key pathological outcome: T cell exhaustion, characterized by progressive loss of function and ineffective viral control. Finally, a vicious cycle is established where the exhausted immune environment and ongoing inflammation further amplify mitochondrial damage, perpetuating the entire process. (cGAS, cyclic GMP-AMP synthase; STING, stimulator of interferon genes; NLRP3, NOD-, LRR- and pyrin domain-containing protein 3; ROS, reactive oxygen species). Figures were created with BioRender.com.

Thus, mtDNA is both a target of HIV-induced damage and a central mediator of downstream immunometabolic dysfunction. Its impaired integrity underlies the bioenergetic collapse that compromises immune function, while its release perpetuates the chronic inflammation characteristic of progressive HIV disease. A unified model integrating these concepts is proposed in **Figure 3**.

## 5 The double-edged sword: antiretroviral therapy and mtDNA

Antiretroviral therapy (ART) represents one of the most successful medical interventions in modern history, transforming HIV infection from a fatal diagnosis into a manageable chronic condition. However, the life-saving benefits of ART come with a metabolic cost, particularly for mitochondrial DNA (mtDNA). The relationship between ART and mtDNA is a classic double-edged sword: while suppressing viral replication alleviates direct viral toxicity to mitochondria, certain drug classes can induce mitochondrial dysfunction, contributing to long-term comorbidities.

### 5.1 Mitochondrial toxicity of early and contemporary ART drugs

The mitochondrial toxicity of ART drugs is primarily linked to their mechanism of action and off-target effects. Among these, nucleoside/nucleotide reverse transcriptase inhibitors (NRTIs) exhibit the most well-characterized mitochondrial toxicity through inhibition of DNA polymerase γ (Pol-γ) (100). These drugs require intracellular phosphorylation to their active triphosphate forms, which then compete with endogenous deoxynucleotides (dNTPs) for incorporation into nascent DNA chains. Although Pol-γ has relatively low affinity for these analogues (23, 100), their incorporation causes premature chain termination during mtDNA replication, leading to mtDNA depletion, reduced expression of -encoded OXPHOS subunits, and ultimately impaired ETC function and ATP production (101–103). Early NRTIs such as Zidovudine (AZT), Stavudine (d4T) and Didanosine (ddI) had a higher affinity for Pol-γ and consequently produced substantial mitochondrial toxicities, including lactic acidosis, hepatic steatosis, peripheral neuropathy, and lipoatrophy (104, 105). In contrast, contemporary NRTIs, such as Tenofovir Alafenamide (TAF), Abacavir (ABC), Lamivudine (3TC), have a much lower affinity for Pol-γ, significantly improving mitochondrial safety profile. TAF, in particular, has largely replaced Tenofovir Disoproxil Fumarate (TDF) due to its superior renal and bone safety and minimal measurable effects on mtDNA (106).

Other antiretroviral classes, including non-nucleoside reverse transcriptase inhibitors (NNRTIs) and protease inhibitors (PIs), do not directly inhibit Pol-γ but are still associated with mitochondrial dysfunction through alternative mechanisms (107, 108). These may include induction of oxidative stress, impairment of antioxidant defenses, inhibition of adipocyte differentiation, promotion of lipolysis contributing to lipodystrophy and metabolic syndrome, dysregulation of glucose metabolism, insulin resistance, and direct disruption of mitochondrial membranes properties or bioenergetic function (100). For example, some PIs have been shown to alter mitochondrial membrane potential and promote ROS production in hepatocytes and adipocytes. Conversely, integrase strand transfer inhibitors (INSTIs) such as Dolutegravir and Raltegravir are generally considered to have the most favorable mitochondrial safety profile, with little evidence of direct mtDNA toxicity. Their main adverse effects, including weight gain and neuropsychiatric symptoms, are not primarily linked to classical mitochondrial pathways (106, 109). Notably, despite effective viral suppression, cellular respiration remains reduced in many immune cells from individuals with chronic HIV infection, and ART is unable to fully reverse this respiratory impairment (110).

### 5.2 ART-induced mtDNA mutations and their clinical implications

Beyond acute depletion, long-term ART exposure is associated with the accumulation of mtDNA mutations, which have profound implications for aging and age-related diseases. The initial insult of Pol-γ inhibition by NRTIs not only depletes mtDNA but can also introduce replication errors, leading to point mutations and large-scale deletions such as the 4977-bp “common deletion” (23, 111). Over time, mutated mtDNA molecules can undergo clonal expansion, and once the mutational burden exceeds a critical threshold—typically 60% and 90% – they produce a biochemical defect in OXPHOS, a concept referred to as the “mtDNA mutational load” (112).

This accumulation mirrors physiological aging. In people living with HIV (PLWH), even under virological suppression, studies report a higher frequency of mtDNA mutations than in age-matched HIV-negative individuals (111, 113). This suggests that ART, in combination with residual HIV-induced inflammation, may contribute to accelerated biological aging(“inflammaging”), potentially underlying the increased risk of age-related comorbidities such as neurocognitive decline, cardiovascular disease, and frailty observed in PLWH (22, 114).

mtDNA integrity is essential for supporting the high metabolic demands of immune cell proliferation and effector function (115). Accumulated mtDNA damage and mutations in immune cells may contribute to incomplete immune reconstitution, wherein some individuals fail to fully cover CD4+ T cell counts despite virological suppression– a process potentially linked to mitochondrial insufficiency (57, 116, 117). Mitochondrial dysfunction also promotes immune senescence, as T cells with impaired mitochondria exhibit features of senescence and exhaustion, characterized by increased expression of inhibitory receptors such as PD-1 and reduced proliferative capacity (56). Additionally, the release of mtDNA as a DAMPs from damaged mitochondria may perpetuate a chronic inflammatory that persists even during ART (115, 118, 119).

Although not yet used in routine clinical practice, assessing mtDNA integrity remains valuable in research setting. Key metrics include mtDNA copy number (reflecting depletion), mtDNA deletion frequency measured through long-fragment PCR or digital PCR, and levels of oxidized mtDNA bases like 8-oxo-dG (111, 120). These biomarkers provide mechanistic insight into long-term ART toxicities and underscore the importance of selecting regimens with minimal mitochondrial impact. The increasing adoption of integrase inhibitor–based therapy represents an important step toward mitigating these risks (22, 114).

## 6 Clinical relevance and translational potential of mtDNA monitoring

The growing understanding of mtDNA’s role in HIV pathogenesis and ART-related toxicity has stimulated interest in its value as a clinically actionable biomarker. However, translating mtDNA measurements into routine practice requires overcoming substantial methodological challenges (121). Key mtDNA parameters—copy number, deletion burden, and mutation load—are highly sensitive to both pre-analytical and analytical variables. mtDNA copy number, deletion burden, and mutation load differ markedly across sample types—including PBMCs, sorted immune subsets, adipose or neural tissues, and biofluids—reflecting the compartment-specific nature of mitochondrial biology (122–126). Pre-analytical factors such as cell isolation procedures, storage conditions, and DNA extraction methods further introduce variability. Analytical platforms also differ in sensitivity and quantification accuracy: quantitative PCR (qPCR) depends heavily on the choice of a stable single-copy nuclear reference gene, whereas digital PCR (dPCR) provides absolute quantification with distinct sensitivity characteristics (127–130). Likewise, methods used to assess mtDNA mutations and deletions—including long-range PCR, NGS, and targeted mitochondrial arrays—vary widely in their ability to detect low heteroplasmy or large structural variants (131–133). Moving forward, establishing consensus protocols for sample processing, assay selection, data normalization, and reporting standards is essential for validating mtDNA-based biomarkers. Such harmonization will enable reliable cross-study comparisons and support the development of clinically meaningful thresholds to guide patient management in people living with HIV.

### 6.1 mtDNA as a biomarker for ART toxicity, metabolic complications, and immunological failure

Quantifying mtDNA damage provides a mechanistic insight into ART-related toxicities and may support personalized management. For instance, reduced mtDNA copy number in subcutaneous adipose tissue is a well-documented feature of the lipoatrophy caused by older NRTIs like stavudine (d4T) and zidovudine (AZT), as mtDNA depletion impairs adipocyte differentiation and triglyceride storage. This measurement remains a valuable marker for assessing residual toxicity of past regimens and for monitoring metabolic health in individuals on contemporary ART, as it correlates with dyslipidemia and insulin resistance (105, 134, 135).

Peripheral neurotoxicity induced by early NRTIs has also been linked to mitochondrial damage and mtDNA depletion in neural tissue. Although mtDNA content in peripheral blood mononuclear cells (PBMCs) has been proposed as an accessible surrogate marker and shows associations with neurological outcomes in some studies, evidence from other cohorts indicates that blood-derived mtDNA does not always mirror nerve tissue changes. Therefore, PBMC mtDNA should be considered a useful but limited biomarker, whose interpretation ought to be integrated with histological, imaging, or neurophysiological evidence (104, 136, 137). Furthermore, although rare, severe toxicities like lactic acidosis are often preceded by a decline in mtDNA copy number together withrising serum lactate, suggesting that serial monitoring in high-risk individuals could allow earlier detection, although current evidence remains limited (22).

The utility of mtDNA extends to assessing immunological failure and exhaustion. A significant subset of people living with HIV (PLWH) who achieve virological suppression fail to restore normal CD4+ T cell counts, a condition known as incomplete immune reconstitution or immunological non-response (INR). Studies associate this state with lower mtDNA copy number and higher levels of mtDNA mutations in CD4+ T cells (116, 117), suggesting an underlying mitochondrial incapacity to fuel the energy-demanding processes of cell proliferation and survival. This supports the position of mtDNA content as a potential predictor of immune recovery success. Similarly, the bioenergetic deficit driven by mtDNA dysfunction is a fundamental driver of T cell exhaustion. A low mtDNA copy number in T cells correlates with increased expression of inhibitory receptors (e.g., PD-1), reduced proliferative capacity, and impaired effector function, thereby offering a metabolic biomarker for the dysfunctional immune state that persists despite ART (22, 116).

### 6.2 Associating mtDNA integrity with comorbidities and disease progression

Longitudinal studies are increasingly linking mtDNA integrity to the risk of developing non-AIDS-defining comorbidities, positioning mitochondrial decline as a central mechanism in HIV-associated accelerated aging. This connection is particularly evident in neurological conditions like HIV-associated neurocognitive disorders (HAND), where mtDNA dysfunction is implicated in pathogenesis. For instance, elevated levels of cell-free mtDNA in the cerebrospinal fluid (CSF) serve as a marker of neuronal damage and mitochondrial stress within the central nervous system. Furthermore, the presence of large mtDNA deletions in neuronal tissue has been linked to HIV sensory neuropathy (136, 138–140).

The correlation between mtDNA copy number in PBMCs and cognitive performance suggests that systemic mitochondrial health reflects processes within the brain. This is supported by population-based prospective cohort studies that showing a significant association between HIV infection and reduced mtDNA copy number in peripheral blood (141), offering a potential less-invasive tool for assessing neurological risk (136, 141, 142). However, interpreting mtDNA changes requires caution, as a decline in mtDNA copy number may also reflect past nucleoside reverse-transcriptase inhibitor toxicity (104). Interestingly, even in patients with an excellent virological response to long-term antiretroviral therapy, such as HIV-infected children, the accumulation of mtDNA mutations persists (143), highlighting the long-term impact of the virus or treatment on mitochondrial genetics.

In the context of cardiovascular disease, chronic inflammation and oxidative stress—key drivers of atherosclerosis—are exacerbated by the release of mtDNA fragments into the bloodstream. These fragments act as pro-inflammatory DAMP, contributing to endothelial dysfunction and plaque instability (68). Lower leukocyte mtDNA copy number and elevated circulating cell-free mtDNA have each been associated with increased cardiovascular events and worse vascular markers in cohort studies and meta-analyses (144–146).

Moreover, the accumulation of somatic mtDNA mutations— a recognized feature of biological aging—appears to be accelerated in PLWH. Studies have reported a higher mutational burden in blood and possibly other tissues of PLWH compared with age-matched controls. This accelerated mtDNA aging phenotype is associated with frailty, multimorbidity, and potentially shorter lifespan (125, 147, 148). Monitoring the “mtDNA mutational load” over time could thus provide a promising biomarker for identifying individuals at elevated risk for age- associated complications, enabling targeted interventions (148). The associations between mtDNA parameters and clinical contexts are summarized in **Table 1**.

**Table 1:** Summary of clinical translation.

Clinical context	mtDNA parameter	Association & potential utility
ART Toxicity	Copy Number (depletion)	Predicts and diagnoses lipoatrophy, neuropathy, lactic acidosis; monitors metabolic health.
Immune Reconstitution	Copy Number (depletion); Mutations	Predicts poor CD4+ recovery (INRs); serves as a biomarker of T cell exhaustion and functional capacity.
Neurocognitive Decline	CSF cell-free mtDNA; Deletions	Marker of neuronal mitochondrial damage; potential diagnostic/prognostic tool for HAND.
Cardiovascular Risk	Circulating cell-free mtDNA	Indicator of systemic inflammation and mitochondrial damage; potential predictor of atherosclerotic events.
Accelerated Aging	Mutational Load (deletions, SNPs)	Biomarker of biological vs. chronological age; identifies individuals at high risk for frailty and age-related comorbidities.

Mitochondrial DNA monitoring thus extends beyond its research role to offer clinical insight. It provides a biological basis for the toxicities and comorbidities in PLWH, moving from correlation toward causation. While standardized clinical assays are still needed, integrating mtDNA metrics into patient management holds promise for advancing personalized medicine in HIV care, shifting the focus from viral suppression to the optimization of metabolic and immunological health

## 7 Therapeutic strategies targeting mitochondria and mtDNA

The recognition of mitochondrial dysfunction as a cornerstone of HIV pathogenesis and ART-related toxicity has catalyzed the search for therapeutic strategies aimed at preserving or restoring mitochondrial health. These approaches range from repurposing existing metabolic modulators and antioxidants to developing gene-editing technologies, offering a promising adjunctive avenue to improve the long-term well-being of PLWH.

### 7.1 Current mitoprotective and metabolic interventions

Research into strategies for protecting mitochondrial integrity in PLWH is exploring several pathways, including the mitigation of oxidative stress, the augmentation of NAD+ levels, and the modulation of key metabolic pathways, often using compounds with established safety profiles.

Antioxidant therapies represent one key approach. This includes agents like Coenzyme Q10 (CoQ10) and its synthetic analogue idebenone. As an electron carrier in the ETC and a lipid-soluble antioxidant, CoQ10 supplementation aims to combat oxidative stress and improve ETC efficiency, with small-scale studies evaluating its potential to ameliorate ART-related fatigue, mitochondrial myopathy, and neuropathy (149–151). Another candidate, N-acetylcysteine (NAC), serves as a precursor to glutathione, the body’s primary antioxidant. By replenishing glutathione stores, NAC helps neutralize ROS and reduce oxidative damage to mtDNA and mitochondrial membranes, showing promise in reducing systemic inflammation and immune activation in PLWH (152, 153).

A second major strategy involves NAD+ augmentation. HIV infection and ART can deplete cellular NAD+, a critical coenzyme for sirtuin activity and energy metabolism. Since sirtuins like SIRT1 and SIRT3 are essential for PGC-1α-mediated mitochondrial biogenesis and antioxidant defense, supplementation with NAD+ precursors such as niacin, nicotinamide riboside (NR), and nicotinamide mononucleotide (NMN) aims to boost NAD+ levels, activate sirtuins, and enhance mitochondrial function. This approach holds promise for countering aging-related phenotypes and metabolic complications (154, 155). However, clinical evidence specifically in PLWH remains very limited, and the optimal dosing, long-term safety, and potential interactions with ART require systematic evaluation.

Investigations into metabolic modulators are also underway. The AMPK activator metformin inhibits mTOR signaling and promotes oxidative metabolism over glycolysis. This action may counter the aberrant glycolytic shift observed in immune cells, potentially alleviating T cell exhaustion and improving insulin sensitivity, leading to clinical trials assessing its impact on inflammation and comorbidities (156–158). However, its immunometabolic effects in ART-treated PLWH remain insufficiently characterized, and observed benefits appear to vary across clinical settings. In addition, gastrointestinal intolerance and variable metabolic responses may limit its broader applicability (156). Similarly, peroxisome proliferator-activated receptor (PPAR)-α agonists such as fenofibrate promote fatty acid oxidation, which may help clear the lipid accumulation associated with mitochondrial dysfunction and reduce inflammation-driven atherosclerosis (159). PPAR agonists also modulate immune responses and enhance antioxidant defenses, positioning them as promising therapies for neurological disorders including Alzheimer’s disease, Parkinson’s disease and HIV-associated neurocognitive disorders (HAND) (160).

Beyond metabolic modulation, more targeted approaches are in development. Mitochondria-specific antioxidants, such as elamipretide (SS-31) represent an innovative strategy. This compound selectively accumulates in the inner mitochondrial membrane, stabilizing cardiolipin to reduce electron leak and ROS production. Having shown efficacy in primary mitochondrial diseases, it is a compelling candidate for addressing HIV/ART-induced mitochondrial damage (161–163).

Finally, ART regimen optimization remains a foundational intervention. The ongoing phased replacement of older, more toxic NRTIs (e.g., d4T, AZT) with newer agents such as tenofovir alafenamide (TAF) and integrase strand transfer inhibitors (INSTIs), which have minimal impact on mtDNA, is a critical step in mitigating iatrogenic mitochondrial harm (164–166).

### 7.2 Future directions: gene editing and precision medicine for mtDNA

The future of mitochondrial medicine lies in moving beyond symptom management to directly target the genetic root of dysfunction—the mtDNA itself. A key direction involves developing gene editing technologies specifically for mutant mtDNA. For instance, mitochondrial-targeted zinc-finger nucleases (mtZFNs) and transcription activator-like effector nucleases (mtTALENs) are engineered to bind specific mtDNA mutation sites. Once inside mitochondria, they create double-strand breaks in the mutant mtDNA, leading to its degradation and selectively enriching wild-type mtDNA, thereby lowering the mutational load below the pathogenic threshold. This approach offers high specificity for known point mutations (167, 168). A more recent advance is mtDNA base editing, which utilizes engineered deaminase enzymes (e.g., DddA-derived cytosine base editors) to catalyze C•G to T•A base conversions in double-stranded mtDNA without creating double-strand breaks. This represents a significant step forward in correcting point mutations, although delivery challenges remain (169, 170). Additional hurdles include the risk of off-target effects in mtDNA or the nuclear genome, potential immune responses to the editing machinery, and substantial technical and regulatory barriers that complicate translation of *in vivo* gene editing into clinical practice. As a result, these approaches remain confined to the preclinical proof-of-concept stage. Beyond editing, strategies are being developed to selectively remove large portions of mutated mtDNA by reconstituting double-strand break repair pathways within mitochondria, potentially eliminating deletion-bearing genomes (171).

Looking ahead, HIV management will increasingly integrate mitochondrial health into a precision medicine framework. This may involve mtDNA biomarker-guided ART selection, where screening for pre-existing mtDNA haplogroups or mutational burdens identifies individuals at higher risk for specific ART toxicities, enabling proactive selection of less harmful regimen (e.g., avoiding certain NRTIs in susceptible individuals). Furthermore, monitoring intervention efficacy through the serial measurement of mtDNA copy number, deletion frequency, and circulating cell-free mtDNA levels will be essential for evaluating mitoprotective therapies in clinical trials and practice. Given the complexity of mitochondrial dysfunction, future strategies may emphasize adjunctive combination therapies—a personalized “mito-cocktail” combining NAD+ precursors to boost biogenesis, mitochondria-targeted antioxidants to reduce stress, and metabolic modulators to optimize fuel utilization, all tailored to individual mitochondrial phenotype.

The therapeutic landscape for mitochondrial dysfunction in HIV is rapidly evolving. While current strategies focus on metabolic support and damage mitigation, the horizon includes transformative technologies capable of directly editing the mitochondrial genome. Integrating these approaches promises to shift HIV care from merely controlling viral replication to comprehensively restoring cellular health and mitigating accelerated aging. An overview of current and future therapeutic strategies is provided in **Table 2**.

**Table 2:** Summary of therapeutic strategies.

Strategy	Mechanism of action	Stage of development	Goal	Challenges/limitations
ART Optimization	Use of TAF, INSTIs over older NRTIs	Standard of Care	Prevent iatrogenic mtDNA damage	Cannot reverse pre-existing damage; newer drugs may have other non-mitochondrial side effects (e.g., weight gain).
Antioxidants (NAC, CoQ10)	Scavenge ROS, boost glutathione	Clinical Research	Reduce oxidative stress & alleviate symptoms (e.g., fatigue)	Often poor bioavailability; limited and inconsistent clinical efficacy data in HIV population.
NAD+ Precursors (NR/NMN)	Activate SIRTs, enhance biogenesis & mitophagy	Preclinical/Early Clinical Research	Improve metabolic health, counter aging	Very limited human data in PLWH; optimal dosing unknown; long-term safety and cost concerns.
Metabolic Modulators (Metformin)	Activate AMPK, promote OXPHOS	Clinical Trials	Reduce inflammation, improve immune function, manage metabolism	Effects in HIV are not fully proven; can cause gastrointestinal intolerance; risk of vitamin B12 deficiency.
Mito-Targeted (Elamipretide)	Stabilizes cardiolipin, reduces ROS	Clinical Research (for other indications)	Improve ETC efficiency, protect mitochondria	Not yet studied in HIV; requires parenteral administration; high development cost.
Gene Editing (mtZFN, Base Editors)	Selective destruction/correction of mutant mtDNA	Preclinical Proof-of-Concept	Cure mtDNA mutations at their genetic source	Immature *in vivo* delivery; risk of off-target effects; major safety and regulatory hurdles.

## 8 Conclusion and future perspectives

**Figure 4:**
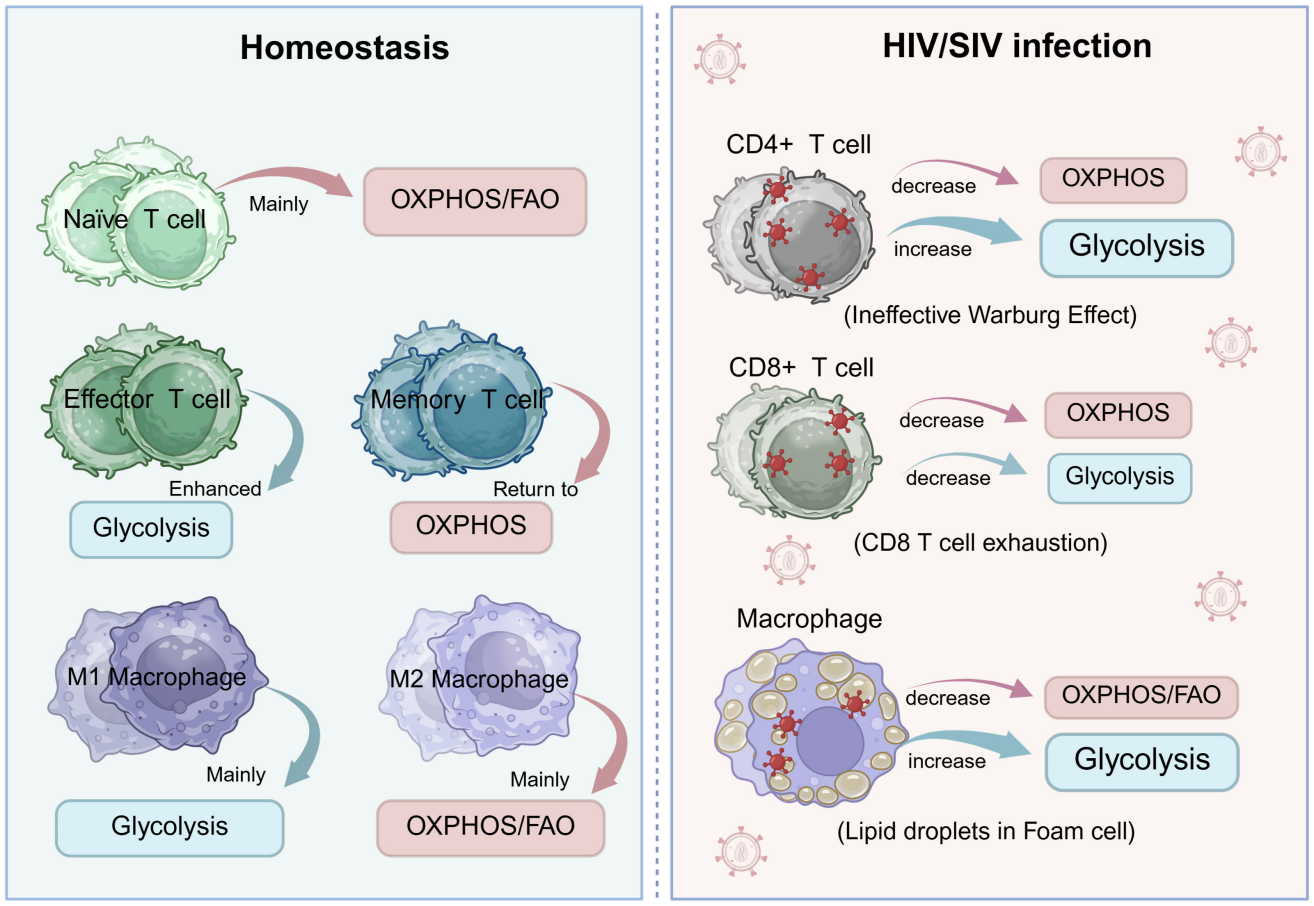
The vicious cycle of mtDNA damage, immunometabolic dysfunction, and chronic inflammation in HIV Infection and antiretroviral therapy. This schematic model illustrates the central role of mitochondrial DNA (mtDNA) damage in driving a self-perpetuating cycle of disease pathogenesis in people living with HIV. The cycle is initiated by two primary insults: 1) HIV/SIV infection, particularly through viral proteins Vpr and Tat, which directly impair mitochondrial function; and 2) Antiretroviral therapy (ART), notably nucleoside reverse transcriptase inhibitors (NRTIs), which inhibit DNA polymerase γ (Pol-γ). These insults trigger mitochondrial damage, characterized by membrane depolarization (ΔΨm↓), elevated reactive oxygen species (ROS) production, and impaired dynamics (increased fission, decreased fusion). A critical consequence is the release of mtDNA into the cytosol. Cytosolic mtDNA then propagates two major downstream pathways: (Left Pathway) Bioenergetic Crisis: Impaired oxidative phosphorylation (OXPHOS) leads to a severe depletion of cellular ATP, depriving immune cells (e.g., CD8+ T cells) of the energy required for proliferation and effector functions, thereby driving T cell exhaustion. (Right Pathway) Innate Immune Activation: mtDNA is recognized as a damage-associated molecular pattern (DAMP) by cytosolic sensors. It activates the cGAS-STING pathway, leading to the production of type I interferons (IFNs), and the NLRP3 inflammasome, resulting in the cleavage and secretion of the pro-inflammatory cytokines IL-1β and IL-18. The convergence of bioenergetic failure and chronic inflammation establishes a state of persistent immune dysfunction. Finally, this inflammatory milieu and the exhausted immune environment feedback to exacerbate mitochondrial damage, thereby closing and perpetuating the vicious cycle that underlies immunometabolic complications and accelerated aging observed in HIV infection despite ART. Figures were created with BioRender.com.

Mitochondrial dysfunction, particularly mtDNA damage, has emerged as a central nexus linking HIV/SIV infection, ART toxicity, and the pervasive state of immunometabolic exhaustion and chronic inflammation that defines the modern HIV management. This review has synthesized evidence showing that mtDNA is both a key victim of viral and iatrogenic insults and a potent mediator of disease pathogenesis. Its damage initiates a vicious cycle: impaired OXPHOS leads to bioenergetic failure and excessive ROS production, which in turn causes further mtDNA damage and release. Cytosolic mtDNA then acts as a persistent trigger of innate immune pathways (e.g., cGAS-STING, NLRP3), fueling chronic inflammation that drives accelerated aging and non-AIDS comorbidities despite virological suppression. As summarized in the proposed model (**Figure 4**), mtDNA damage lies at the core of a self-perpetuating cycle that connects early viral and drug insults to the chronic immunometabolic dysfunction that persists throughout HIV infection.

Translating this mechanistic understanding into clinical practice is paramount. Monitoring mtDNA copy number, deletion frequency, and mutation load offers a window into cellular metabolic health, with potential as biomarkers for predicting ART toxicity, assessing immune reconstitution, and stratifying risk for neurological, cardiovascular, and age-related complications. While current strategies focuses on mitigation—using antioxidants, NAD+ precursors, and metabolic modulators to support mitochondrial function—the future lies in targeted, potentially curative interventions. Mitochondrial gene editing technologies, such as base editors and engineered nucleases, heralds a new era in which correcting or eliminating pathogenic mtDNA mutations becomes feasible.

Looking forward, several key research directions warrant urgent investigation:

Spatiotemporal Dynamics of mtDNA Damage: Future studies should move beyond bulk tissue to map cell-type specific mtDNA mutations (e.g., exhausted CD8+ T cells vs. tissue-resident macrophages) using single-cell technologies. Employing digital PCR (dPCR) and next-generation sequencing to longitudinally quantify mtDNA mutations in paired peripheral blood, tissue biopsies, and CSF will clarify their roles in residual inflammation, neurocognitive decline, and immunological failure.

Causal Role of mtDNA in Inflammation and Exhaustion: Mechanistic studies are needed to test whether mtDNA deletion mutations directly drive cGAS-STING activation in chronic HIV infection. This involves isolating immune cells with high mutational loads and specifically inhibiting this pathway to assess its contribution to the IFN signature and T cell exhaustion.

Interventional Clinical Trials: Well-designed trials are urgently needed to evaluate mitoprotective adjuvants (e.g., NMN/NR, elamipretide, metformin) in PLWH. These studies should use mtDNA health as a primary endpoint (e.g., copy number, deletion load), alongside measures of immune function, inflammation, and clinical outcomes, to establish a direct link between improved mitochondrial function and patient health.

Gene Editing for HIV-Related mtDNA Disease: The feasibility of using mitochondrial base editors to correct ART-induced point mutations or eliminate deleted genomes should be explored in preclinical models. This represents the ultimate precision medicine approach to eradicating the genetic legacy of past drug toxicities.

In conclusion, viewing HIV/AIDS through the lens of mitochondrial health provides a unifying framework for its complex pathophysiology. By focusing on restoring mitochondrial integrity– through supportive care, precision medicine, or genetic correction–we can envision a future where HIV treatment goals extend beyond undetectable viral loads to encompass full metabolic and immunological rejuvenation, granting people living with HIV not only longer but healthier lives.
